# Investigating the Variation of Benzene and 1,3-Butadiene in the UK during 2000–2020

**DOI:** 10.3390/ijerph191911904

**Published:** 2022-09-21

**Authors:** Rayne Holland, M. Anwar H. Khan, James C. Matthews, Sophia Bonifacio, Rhian Walters, Priya Koria, Joanna Clowes, Karla Rodgers, Temi Jones, Leeya Patel, Rhianna Cross, Freya Sandberg, Dudley E. Shallcross

**Affiliations:** 1 School of Chemistry, University of Bristol, Bristol BS8 1TS, UK; 2 Department Chemistry, University of the Western Cape, Robert Sobukwe Road, Bellville 7535, South Africa

**Keywords:** volatile organic compounds, urban areas, rural areas, air quality strategy, seasonal variation, cancer impact

## Abstract

The concentrations of benzene and 1,3-butadiene in urban, suburban, and rural sites of the U.K. were investigated across 20 years (2000–2020) to assess the impacts of pollution control strategies. Given the known toxicity of these pollutants, it is necessary to investigate national long-term trends across a range of site types. We conclude that whilst legislative intervention has been successful in reducing benzene and 1,3-butadiene pollution from vehicular sources, previously overlooked sources must now be considered as they begin to dominate in contribution to ambient pollution. Benzene concentrations in urban areas were found to be ~5-fold greater than those in rural areas, whilst 1,3-butadiene concentrations were up to ~10-fold greater. The seasonal variation of pollutant concentration exhibited a maximum in the winter and a minimum in the summer with summer: winter ratios of 1:2.5 and 1:1.6 for benzene and 1,3-butadiene, respectively. Across the period investigated (2000–2020), the concentrations of benzene decreased by 85% and 1,3-butadiene concentrations by 91%. A notable difference could be seen between the two decades studied (2000–2010, 2010–2020) with a significantly greater drop evident in the first decade than in the second, proving, whilst previously successful, legislative interventions are no longer sufficiently limiting ambient concentrations of these pollutants. The health impacts of these pollutants are discussed, and cancer impact indices were utilized allowing estimation of cancer impacts across the past 20 years for different site types. Those particularly vulnerable to the adverse health effects of benzene and 1,3-butadiene pollution are discussed.

## 1. Introduction

Volatile organic compounds (VOCs) are a complex mixture of molecules that are emitted into the atmosphere from numerous natural and anthropogenic sources [1]. Of the VOCs, benzene (C_6_H_6_) and 1,3-butadiene (C_4_H_6_) are of particular importance because they are understood to be amongst the most toxic [2]. 

Benzene pollution results from domestic heating, waste incineration, evaporation of petroleum, and incomplete combustion of fossil fuels, namely in road transport [3,4,5,6]. Historically, road traffic was the most significant contributor to benzene pollution in the UK. However, as the target of most mitigation legislation, the contribution of this source has significantly decreased with domestic sources now the largest contributor at ~30% [7]. Similarly, 1,3-butadiene is now most significant in domestic environments, though vehicle emissions and industry also remain significant sources [7,8].

Once released into the atmosphere, benzene and 1,3-butadiene degrade by OH oxidation with an associated rate constant at 298 K of 1.22 × 10^−12^ and 66.6 × 10^−12^ cm^3^ molecule^−1^ s^−1^, respectively [9]. Typical lifetimes for benzene and 1,3-butadiene differ due to variability of OH concentration, sunlight intensity, and temperature but are of the order of 10 days and 4 h, respectively. For 1,3-butadiene, in polluted environments, additional loss through reaction with NO_3_ radicals (approx. 25%) is important [10], whereas loss through ozonolysis is slow (2%) but makes a small additional contribution [11]. Away from elevated NO_x_ environments, OH loss will dominate. The degradation occurs through OH addition across the double bonds for 1,3-butadiene and to the benzene ring to form an initial adduct, forming radicals, which subsequently undergo a series of reactions with O_2_ and/or NO forming a mixture of neutral products [9]. Prior to degradation, these pollutants can enter the body primarily through inhalation, causing a multitude of adverse health effects [12,13]. 

Pollutant damage can be acute, resulting from immediate exposure to high pollutant concentrations, or chronic, as a result of prolonged exposure to low 1,3-butadiene and benzene concentrations. Chronic damage is of increasing concern, affecting millions globally and having significant health consequences [1,14,15,16]. Benzene and 1,3-butadiene are defined as Group 1 carcinogens (known to cause cancer), according to the International Agency for Research in Cancer [13,17], as their reactive metabolites can bind to DNA, leading to cancers such as leukemia and haematolymphatic cancer for benzene and 1,3-butadiene, respectively [18,19]. The adverse health effects of these pollutants have been extensively documented and reviewed [14,15,20,21,22]. In particular, occupational exposure to these pollutants remains a focus of many health impact studies [23,24,25,26,27,28]. The main exposure pathway for both benzene and 1,3-butadiene is inhalation, and as such ambient air pollution is likely to be the most significant source of exposure to the majority of the population [3,29].

Owing to the adverse health effects of these pollutants, their presence in the atmosphere has been well documented for years and the cancerous effects of 1,3-butadiene and benzene have been at the forefront of research [3,4,30]. Additionally, research has focused on how exposure to these pollutants varies for different demographics, especially for groups that experience higher exposure levels or are more susceptible to DNA damage [1,18,31,32]. Whilst background concentrations remain a concern, those living in urban areas with concentrated road traffic and other anthropogenic emissions, have historically experienced greater exposure than others. Whilst this remains true, sources that were previously less significant are becoming more important contributors as the influence of road traffic sources decreases with focused legislation. As these sources are likely to also be active in rural environments it is vital to assess their contribution and influence on population exposure.

With the World Health Organization (WHO) estimating 4.2 million premature deaths every year as a direct result of air pollution (with 6% attributed to cancer [33]), coupled with increasing levels of anthropogenic influence globally, monitoring of these pollutants remains important both globally and in the UK to maintain the health of the population. 

Despite a reduction in UK emissions over the past two decades, these compounds remain genotoxic and no safe level of exposure can be defined [3]; therefore, an improved understanding of these species and their public health impact is paramount. In this study, we investigated the trends of benzene and 1,3-butadiene concentrations in the urban, suburban, and rural areas of the UK during the period 2000–2020. Long-term studies such as this are scarce in the literature and are paramount in understanding the changing exposure of the general public to these pollutants and the legislative means by which exposure may be reduced.

## 2. Methods

Mean daily averages for the concentrations of benzene and 1,3-butadiene (with uncertainty of 15%) [34] were obtained from the UK Air Information Resource (UK AIR—https://uk-air.defra.gov.uk/data/ (accessed on 18 January 2022)) from the Department for Environment Food and Rural Affairs (DEFRA) [35]. Concentrations are measured as part of the Automatic Hydrocarbon Network (Automatic Hydrocarbon Network—Defra, London, UK) and all sites utilize Perkin Elmer gas chromatographs to measure a wide range of hydrocarbons [36]. 

Measurement locations chosen included representations of varying environments in the UK including suburban (London Eltham, LE), rural background (Harwell, H, and Auchencorth Moss, AM), and urban traffic (London Marylebone Road, LMR). Data coverage of the sites was inconsistent and individual sites selected were chosen to maximize data coverage for the specified time period and environment type [31].

To illustrate the potential health impacts of concentration changes of these chemicals on cancer incidence, we have utilized recent research into risk indices of leukemias as a result of ambient benzene and 1,3-butadiene concentrations [27,37,38,39,40,41]. We utilize the conclusion that continuous exposure to 1 ppb of benzene causes 2.4 additional deaths in a population of 100,000, and continuous exposure to 1 ppb of 1,3-butadiene causes 3 additional deaths per 100,000 people. The benzene risk estimate was provided by the National Industrial Chemical Notification and Assessment Scheme for leukemia, Australia [37], by extrapolating from occupational exposures from Crump [27] and US EPA [38]. These studies look primarily at follow-up mortality data from an occupationally exposed cohort as opposed to specific biomarker monitoring. Cancer of bone marrow was also considered but found to be low risk. The risk estimate for 1,3-butadiene was calculated by the EPA Integrated Risk Information System [39] derived from a combination of a cohort study of workers exposed to 1,3-butadiene [40] reanalyzed by Health Canada [41] to find a relative risk of leukemia. This risk was adjusted to include total cancer risk through exposure to 1,3-butadiene, justified by the effect of exposure on rodents on mammary cancers. In the same study group, evidence of a link between 1,3-butadiene and lung cancers was weak. Of course, these indices, such as all of this nature, undoubtedly have considerable uncertainty. However, in the absence of UK-based, outdoor ambient exposure studies, these indices are useful for a preliminary investigation on public health impact that may subsequently be used to inform more thorough studies. 

## 3. Results and Discussion

The atmospheric concentrations of 1,3-butadiene are found to be significantly lower than those of benzene across all sites considered between 2000 and 2020. At suburban and urban traffic sites, 1,3-butadiene concentrations are just 13 and 24% of corresponding benzene concentrations, respectively. This decreases further for rural sites which see 1,3-butadiene concentrations at 12% of corresponding benzene concentrations. This is likely due to benzene’s additional sources of pollution (including domestic heating, waste incineration, and evaporative emissions [42]) and longer atmospheric lifetime. 

Observing trends over the 20 years analyzed, all sites showed an overall decrease in concentration for both 1,3-butadiene and benzene (though this was statistically insignificant at the rural sites AM and H site, *p* > 0.05, see Supplementary Information S1–S3). In the cases of LMR and LE, ambient concentration decreases are much more significant in the first decade than in the second (Table 1, Supplementary Information S4 and S5 for graphs). 

Out of all locations studied, LMR showed the most significant reduction in benzene and 1,3-butadiene pollution from 2000–2020 (Table 1). As a kerbside location in the center of London, situated next to a six-lane road with traffic flows of over 80,000 vehicles per day [43], LMR is a monitoring site which is significantly affected by changes in road traffic. Consequently, as the U.K. Government has focused emission reduction initiatives on road transport in the past 20 years, it is not surprising that LMR showed the greatest reduction in benzene and 1,3-butadiene pollution over this period. This also parallels with rural sites (H and AM) showing the insignificant reduction in benzene and 1,3-butadiene pollution over time as both sites are located some distance from any major roads [36]. In fact, AM can be considered a ‘background’ site as a result of its relative stability to the most significant anthropogenic influences.

The rapid decrease at LMR was likely motivated by the introduction of strategy control schemes, such as the introduction of EU legal concentration limits (1.5 ppb for benzene and 1.0 ppb for 1,3-butadiene) [44]. Specifically, Euro 3 and 4 emission standards in 2000 and 2005 required new petrol and diesel vehicles to be equipped with a catalytic converter; these are expected to have contributed to this decline [45]. Previously, fleet technology improvement, such as the introduction of the 1992 catalytic convertors legislation, requiring all petrol vehicles to be fitted with catalytic convertors, which likely also led to ‘cleaner’ emissions [8]. Decreasing trends of other pollutants found in vehicle emissions, such as NO_x_, are consistent with this data, supporting the influence of advanced technology [46]. Furthermore, multiple emission control strategies were introduced in line with the London Mayor’s Air Quality Strategy in 2010, including the implementation of the London Low Emission Zone (LEZ), congestion charging, and diesel–electric hybrid buses [47,48]. There is also a link between the UK government’s tax cut on diesel vehicles’ fuel duty in 2001, which encouraged the switch to diesel cars from petrol, resulting in around 12 million tons of oil equivalent (Mtoe) petrol being replaced with diesel from 1990 to 2017 [49]. With diesel emissions containing significantly fewer hydrocarbons than petrol emissions [8], the introduction of these vehicles likely contributed to lowering levels of these pollutants [50]. As a result, the composition of the national vehicle fleet has significantly changed in the past 20 years, which is a major contributor to the reduced benzene and 1,3-butadiene pollution. Again, the clear focus on and relative success of traffic-related emission mitigation strategies is further supported by the comparatively insubstantial decreases in concentration seen at non-traffic sites for which such measures have a lesser influence.

**Figure 1:**
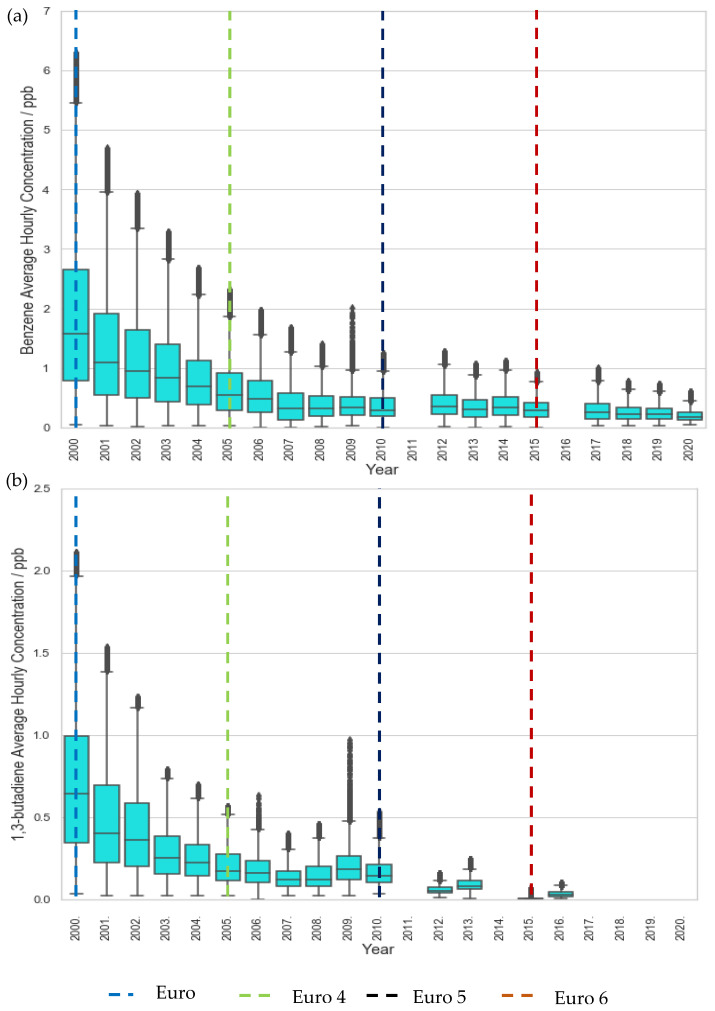
Trends in hourly-average pollutant concentration at the traffic site, LMR over the period 2000–2020 (**a**) benzene and (**b**) 1,3 butadiene. Error bars represent the 95% confidence level in pollutant concentration. Vertical lines represent implementation year of each relevant Euro control. Years with data coverage below 70% were omitted from analysis to avoid seasonal skewing (see Appendix A for data coverage representation).

**Figure 2:**
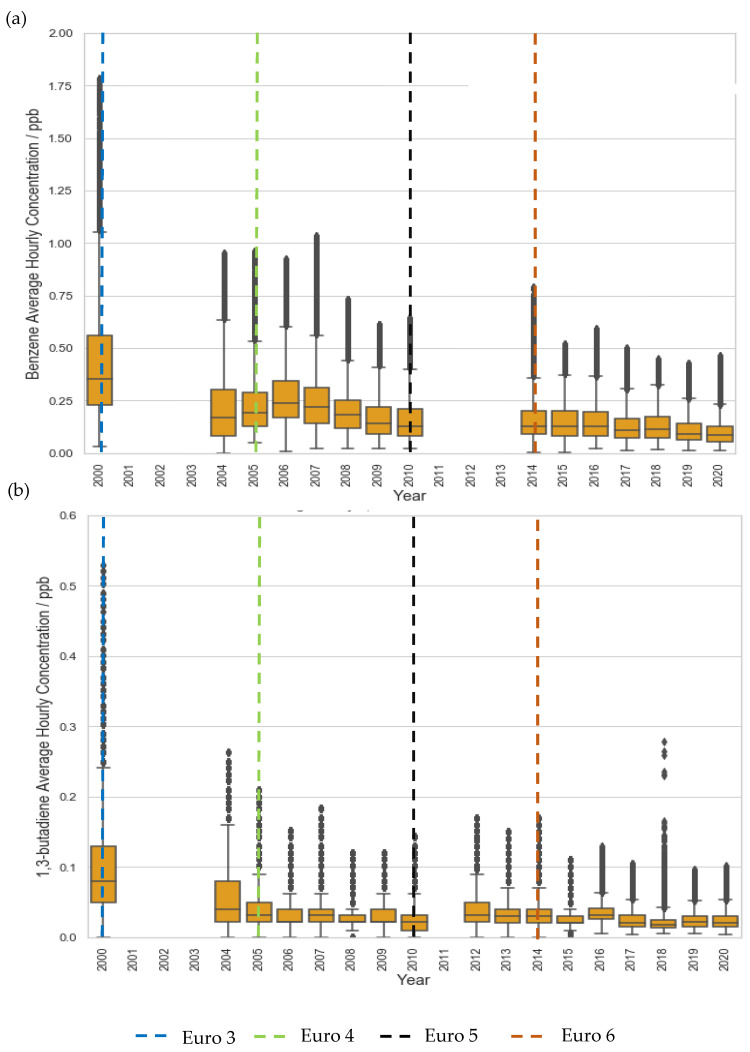
Trends in hourly-average pollutant concentration at the suburban site LE over the period 2000–2020, (**a**) benzene and (**b**) 1,3 butadiene. Error bars represent the 95% confidence level in pollutant concentration. Vertical lines represent implementation year of each relevant Euro control. Years with data coverage below 70% were omitted from analysis to avoid seasonal skewing (see Appendix A for data coverage representation).

**Figure 3:**
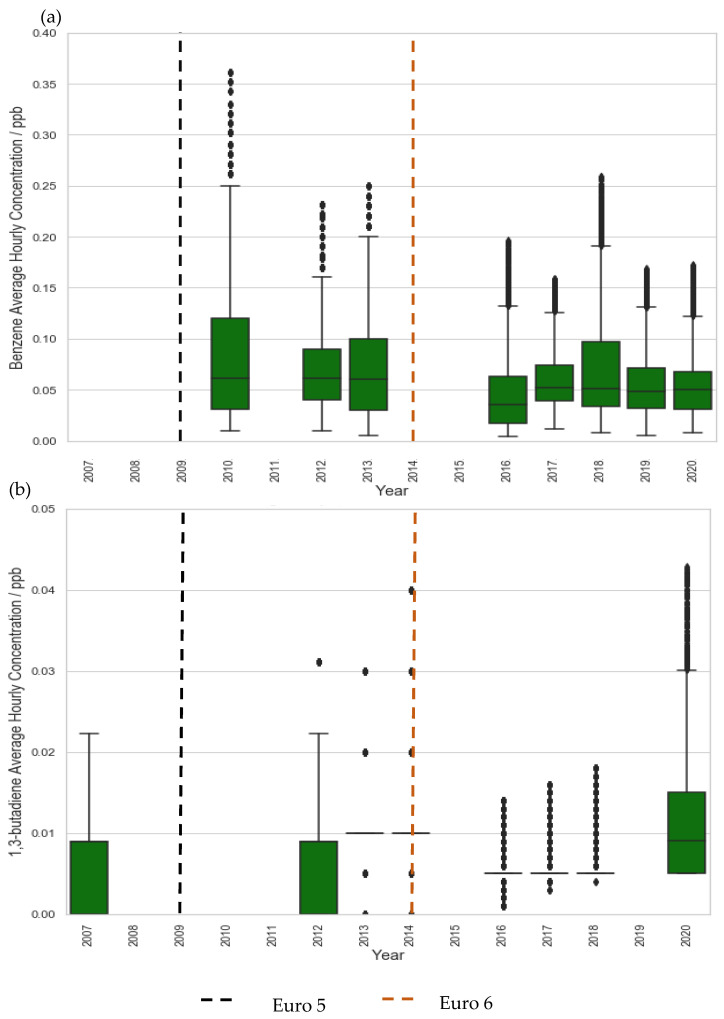
Trends in hourly-average pollutant concentration at the rural site AM over the period 2007–2020 (**a**) benzene and (**b**) 1,3 butadiene. Error bars represent the 95% confidence level in pollutant concentration. Vertical lines represent implementation year of each relevant Euro control. Years with data coverage below 70% were omitted from analysis to avoid seasonal skewing (see Appendix A for data coverage representation).

**Figure 4:**
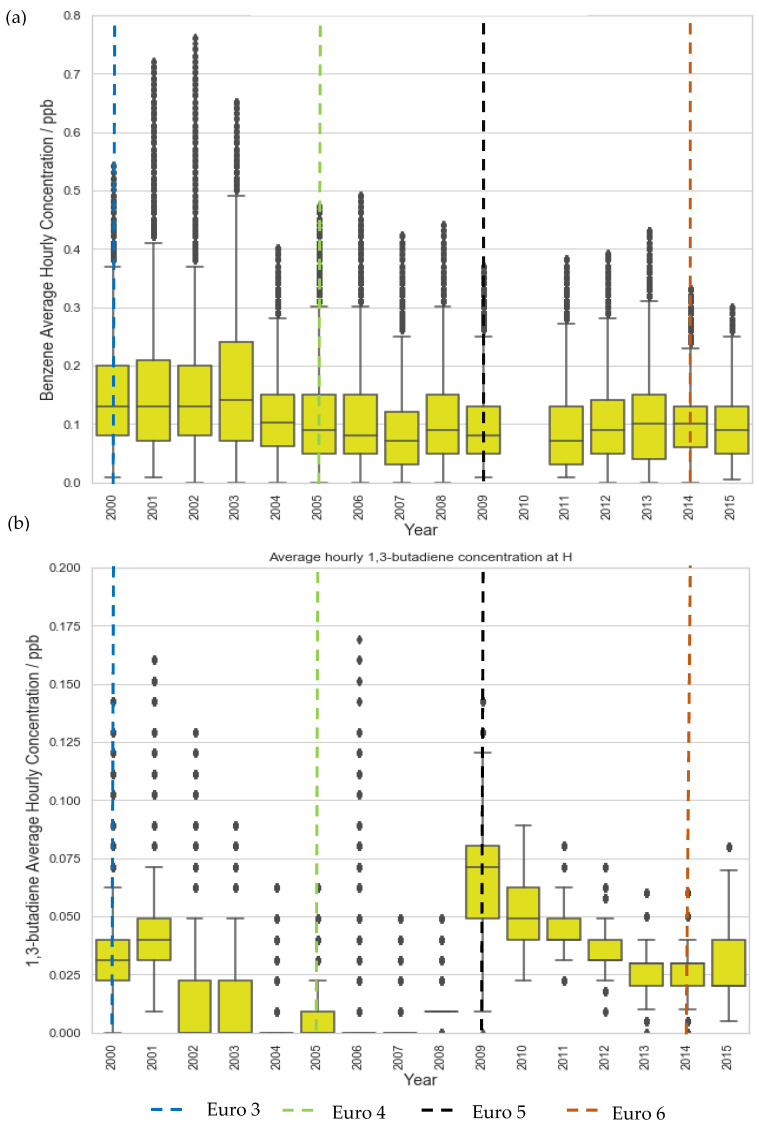
Trends in hourly-average pollutant concentration at the rural site H over the period 2000–2015 (**a**) benzene and (**b**) 1,3 butadiene. Error bars represent the 95% confidence level in pollutant concentration. Vertical lines represent implementation year of each relevant Euro control. Years with data coverage below 70% were omitted from analysis to avoid seasonal skewing (see Appendix A for data coverage representation).

Even at the traffic site, LMR, a plateau in concentration reductions is seen post-2015 (Figure 1). Similarly, benzene and 1,3-butadiene concentration at LE, a suburban site, decreased significantly in the first decade but began to plateau after 2009, eventually converging to levels comparable to rural background sites (Figure 2, Figure 3 and Figure 4). These plateaus can be attributed to background sources that are not being addressed to the same extent under current legislation such as industry, agriculture, and shipping [51,52,53]. To further reduce the remaining pollution and associated cancer risk, these sources need to be addressed and appropriate measures taken to ensure the concentrations continue to decrease. This further illustrates the need for additional measures, perhaps in the context of non-traffic sources, in order to continue reducing the concentrations of these harmful pollutants.

Concentrations at urban traffic site LMR remained noticeably and expectedly greater throughout the period studied due to considerably higher volumes of traffic. Comparison of LMR with other site types showed that the yearly pollutant concentration was on average ~5- and ~10-fold greater for benzene and 1,3-butadiene across the period 2000–2020. Consequently, residents in urban areas are exposed to higher levels of pollution compared with those living in rural areas and are therefore at greater risk of developing associated diseases.

By comparing the sites with data available for the entire 20-year analysis, LMR and LE, a smaller decline in benzene concentration, compared with that of 1,3-butadiene, can be seen. For LMR (2000–2020), benzene concentration declined 89% compared with 92% for 1,3-butadiene; likewise, the suburban site, LE showed 75% and 85% decrease for benzene and 1,3-butadiene, respectively. The greater decrease in 1,3-butadiene could be reflective of vehicle emissions having a higher contribution to overall 1,3-butadiene levels relative to benzene, which has additional sources of pollution, as mentioned previously. Moreover, with a 6% difference between pollutant decrease in the urban traffic site, compared with 10% in the suburban site, it could be reasoned that the slight disparity is partly due to the contribution of other sources of benzene found predominantly in residential areas. These sources have likely not been reduced and now may have a comparably higher contribution than previously. Findings from a study by the UK Health Protection Agency support this, showing that benzene pollution from vehicle emissions had decreased from 60% to 20% by 2004, contributing less than domestic sources, which made up the greatest source of benzene at 33% [3,7]. 

**Figure 5:**
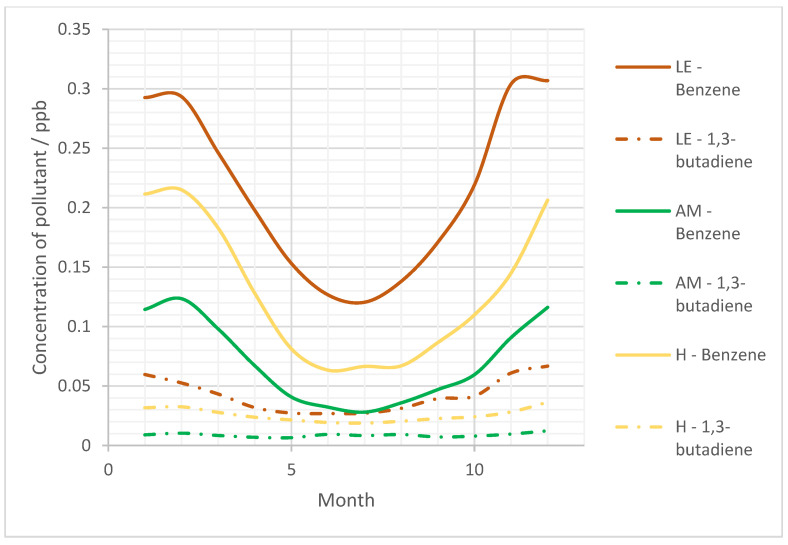
Overall seasonal variation (hourly concentrations averaged across 2000–2020) of each pollutant concentration at each of the non-traffic sites LE, AM, and H. LMR excluded from plot due to significantly elevated concentrations in comparison to the sites presented here; same seasonal trend is seen with increased variability due to increased anthropogenic influence.

Moving forwards, the seasonal variations of the pollutants were considered to indicate the variation of exposure. The seasonal variations in the concentration of benzene and 1,3-butadiene generally show similar trends across all sites, with the maximum concentrations occurring during the winter and the minimum concentrations occurring during the summer in agreement with previous studies [8,9,54]. This seasonality was more pronounced for sites with lower pollution as they experience less anthropogenic influence which may have competing seasonal variations (Figure 5). 

This trend was seen for all years for benzene and 1,3-butadiene, with the exception of 2008, 2009, 2012, and 2020, this was also the case. Records for 2008–2009 1,3-butadiene concentrations showed inversed results where higher levels were observed in summer than in winter. This is an unexpected trend as it does not align with previous observations for other years but could be due to increased tourism during those years or even increased use of cars, further investigations would need to be carried out to source the reason for this observation [55].

These seasonal trends are driven by the oxidative capacity of the atmosphere which varies with OH radical concentration. Summer experiences increased insolation and sunlight intensity [30], leading to increased atmospheric OH (approximately double that of winter) [56]. This causes increased rates of pollutant degradation and hence lower pollutant levels are observed. Additional factors, such as ‘cold starts’ from petrol vehicles have also be found to contribute to higher winter levels of benzene, but are much less significant than OH [30]. 

Interestingly, the seasonality of benzene concentration was more pronounced than that of 1,3-butadiene. In a few instances (2004, 2005, 2009, and 2010), winter levels of benzene were 2-fold higher than those observed in summer. UK Met. Office meteorological information shows that 2003–2005 had some turbulent weather with strong winds and high temperatures [57]. These conditions helped to demonstrate the impact of biogenic sources on overall emissions and on the impact of meteorology on observed concentrations [5]. When assuming pollutant degradation rate is similar for all sites, it is likely that higher variation for benzene concentrations is due to changes in source levels throughout the year. A probable explanation is decreased use of domestic heating in summer in which, unlike 1,3-butadiene, benzene is a significant source [3]. This habitual change results in variations of benzene emitted between seasons, whereas source emissions of 1,3-butadiene remains relatively constant [44]. It is also possible that 1,3-butadiene concentrations are less influenced by OH concentration compared with benzene. 1,3-butadiene has additional degradation processes, such as reactions with the nitrate radical (NO_3_) and ozone (O_3_) [8], which are not affected by seasonal changes to the same extent as OH and therefore the removal of 1,3-butadiene between summer and winter is less varied than that of benzene. As a result, the concentration of 1,3-butadiene is less varied across the year.

### Cancer Impact Estimation

Observed reductions in benzene and 1,3-butadiene pollution can be expected to correlate with a reduction in health impact over the same period. Results herein are discussed as additional deaths due to associated cancers of each pollutant, which correlates directly with pollutant concentration, according to risk factors detailed previously.

**Figure 6:**
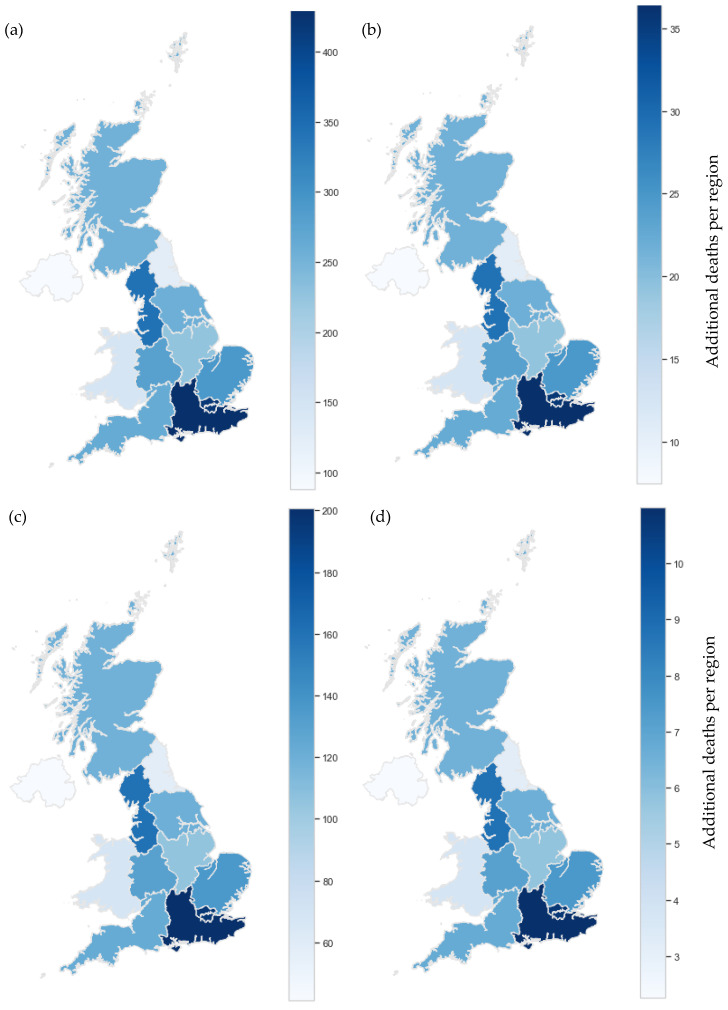
Upper and lower limits of average yearly cancer impact from benzene and 1,3-butadiene for 2000. (**a**) Benzene urban, (**b**) Benzene rural, (**c**) 1,3-Butadiene urban, (**d**) 1,3-Butadiene rural.

**Figure 7:**
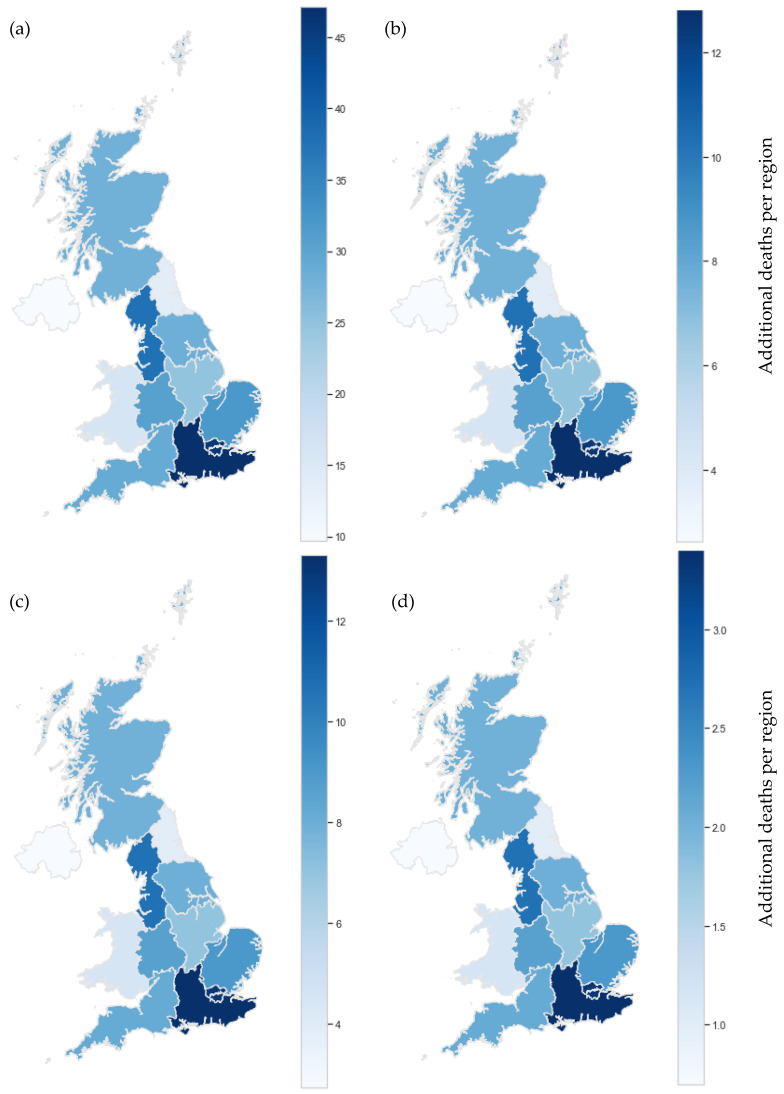
Upper and lower limits of average yearly cancer impact from benzene and 1,3-butadiene for 2020. (**a**) Benzene urban, (**b**) Benzene rural, (**c**) 1,3-Butadiene urban, (**d**) 1,3-Butadiene rural.

Whilst benzene generally exhibits higher concentrations, due to 1,3-butadiene’s higher risk factor the cancer impacts of the two pollutants are generally comparable. For instance, LMR data showed, for 2000, an average of 5 additional deaths per 100,000 for ambient benzene pollution and 2 additional deaths per 100,000 for ambient 1,3-butadiene pollution. Compared with cancer death totals reported by the UK House of Commons Library [58], these values imply ambient concentrations of these pollutants could each account for approximately 1% of all cancer deaths in England. Utilized health indices suggest that 1,3-butadiene is more genotoxic than benzene, supported by reports suggesting 1,3-butadiene is more closely linked to DNA damage [59]. Given its increased potency, it is surprising that, in contrast to benzene, 1,3-butadiene is not covered in EU Directives and there are fewer estimates for the cancer impact of 1,3-butadiene [60]. However, Figure 6 and Figure 7 present results suggesting that urban, ambient 1,3-butadiene concentrations have a similar health effect as rural, ambient concentrations of benzene suggesting benzene control is a more pressing issue in the context of public health.

To represent the upper and lower limit cancer impact of benzene and 1,3-butadiene in the U.K., the average cancer impact at urban and rural sites was paired with population data obtained from the Office for National Statistics (ONS) [49] and plotted on a national map for 2000 and 2020 (Figure 6 and Figure 7). Despite the COVID-19-related lockdowns experienced in 2020, ambient concentrations of benzene and 1,3-butadiene saw no statistically significant changes from 2019 and so are appropriate for use in this analysis.

Whilst the impact per 100,000 is relatively small, it is by no means negligible. In fact, when additional deaths per region are considered, we see a concerning impact in 2000. This is particularly true when average urban concentrations are considered (Figure 6). Given that 78.7% of the total English population were living in classified ‘urban’ areas in 2000 [61], urban estimates are likely to be our best estimation. This percentage has since increased to approximately 82.9% for 2019 and continues to increase annually [62]. In urban areas, sources from industrial processes and vehicle emissions are much higher, explaining the elevated cancer risk. With increasing urbanization, ambient pollution at urban sites is likely to be more representative of the exposure of the general population than ambient pollution at rural sites. Figure 6 visualizes the high levels of mortality associated with benzene and 1,3-butadiene pollution in urban areas of the U.K in 2000. Moving to Figure 7, we see a significant decrease in estimated additional deaths for 2020. However, despite all sites meeting legal limits [44], estimated additional deaths remained non-negligible in the range of 1 to 45 per region for 2020. Furthermore, these values are likely to be an underestimation. Firstly, the basis of these health indices are studies conducted with all male cohorts [27,40]. It is suggested by the US EPA that the index for 1,3-butadiene be doubled to account for effects on women and children [39]. This has not been done in this investigation due to uncertainty concerns with applying this logic to other health indices but certainly merits further investigation. Secondly, this investigation assumes a relationship between continuous ambient pollution exposure and additional cancer deaths. Alternatively, a cumulative representation of ambient pollutant exposure could be considered as it has been in previous literature, including those on which the indices utilized here are based [27,37]. With a cumulative exposure approach, great care is needed to avoid gross overestimation of health impact. Where possible, pharmacokinetic studies, retention estimations and absorption metrics should all be considered. This is beyond the scope of this project and has not been completed at this time, but, again, further investigation would prove valuable. 

A shortcoming in our method comes from the fact that the general population spend the majority of their time indoors. However, recent studies have concluded that indoor ambient pollution of benzene and 1,3-butadiene is strongly associated with outdoor pollution [63,64], and as such our method is fit for purpose as a first approximation of general exposure trends.

It is known that there are no safe levels of benzene nor 1,3-butadiene exposure [1,3]; limits are put in place to minimize the adverse effects of pollutants rather than eliminate cancer risk. However, significant cancer impacts in urban areas demonstrates these approaches are ineffective in minimizing adverse effects sufficiently. Trend analysis presented here has demonstrated that improving and reducing vehicle emissions significantly reduces the risk of cancer. Nevertheless, levels of pollution are still substantial, thus control strategies need to address additional sources to work towards elimination of benzene and 1,3-butadiene and, in turn, protect the health of the nation.

The seasonal trends in concentrations discussed previously suggest higher exposure is likely to be experienced in the winter; it is valuable to consider variational exposure of particularly vulnerable demographics. Particularly important is the consideration of fetal development in pregnant women. Maternal exposure to benzene and 1,3-butadiene emissions in the first trimester of pregnancy can increase incidences of genetic susceptibility to cancer for the fetus [65]. Women pregnant in winter are more exposed to these carcinogenic emissions and thus more at risk to this susceptibility. Additionally, exposure to emissions of benzene and 1,3-butadiene are also reported to cause increased cases of childhood leukemia [65]. These are important considerations and further investigation should be conducted on seasonal exposure variation.

## 4. Conclusions

Decreasing trends in the ambient concentrations of benzene and 1,3-butadiene highlights the success of emission control strategies adopted in the U.K. in reducing benzene and 1,3-butadiene pollution. The large reduction in pollution observed at LMR, between 2000 and 2010, particularly emphasizes the decreasing influence of road traffic on these pollutants. A plateau in the reduction of benzene and 1,3-butadiene pollution from ~2015 implies that the contribution of road transport to emissions has gradually decreased over time and other sources have become dominant. Therefore, it will be beneficial to investigate the different U.K. sources of benzene and 1,3-butadiene in future, to identify the source of constant pollution levels observed since 2015 and determine the industry in which emission reduction initiatives should be targeted. 

Benzene and 1,3-butadiene pollution was notably higher in urban areas compared with rural areas by ~5-fold and ~10-fold, respectively, which highlights the need for geographically directed policies in future. Also, the upper and lower limit cancer impact from these species showed that despite the average rural (lower limit) concentrations of benzene and 1,3-butadiene meeting national air quality objectives, a measurable cancer impact is still likely. Therefore, although national pollution targets are often successful in cutting emissions, they are not effective in terms of protecting public health as no safe level of exposure to these pollutants can be clearly defined. 

It is important to note, the cancer impact indices utilized in this study are not without fault. Epidemiology and exposure relationships are complicated and undoubtedly the indices represent an over-simplification of the true relationship and many confounding factors are ignored. However, this study provides a good first approximation of the likely effect of these pollutants and such effects have varied as a result of legislative intervention. Conclusively, the analysis used in this work could be applied on a larger scale as a means to improve health data and direct future policy development.

## Figures and Tables

**Table 1:** Summary table of graphical analyses for each site across the whole available dataset and split into decadal subsets. Where trend proved to be statistically insignificant (*p* > 0.05), label of ‘Not significant’ was used. N/A label used where data was unavailable for a subset.

Site	Data Availability	Overall Gradient of Line of Best Fit		Gradient of Line of Best Fit for Data 2000–2010		Gradient of Line of Best Fit for Data 2010–2020	
		Benzene	1,3-Butadiene	Benzene	1,3-Butadiene	Benzene	1,3-Butadiene
LMR	2000–2020	−0.062	−0.028	−0.144	−0.047	−0.019	−0.011
LE	2000–2020	−0.013	−0.003	−0.028	−0.009	−0.007	−0.001
AM	2010–2020	−0.003	Not significant	N/A	Not significant	−0.003	Not significant
H	2000–2015	−0.005	Not significant	−0.001	Not significant	−0.001	Not significant

## Data Availability

The data presented in this study are available on request from corresponding author.

## Supplementary Materials

The following supporting information can be downloaded at: https://www.mdpi.com/article/10.3390/ijerph191911904/s1.

## Appendix A

**Table A1:** Representation of data availability for this study. Years where data coverage was less than 50% (red) have been removed to avoid seasonal skewing.

Benzene					1,3-Butadiene				
Year	LMR	LE	AM	H	Year	LMR	LE	AM	H
2000					2000				
2001					2001				
2002					2002				
2003					2003				
2004					2004				
2005					2005				
2006					2006				
2007					2007				
2008					2008				
2009					2009				
2010					2010				
2011					2011				
2012					2012				
2013					2013				
2014					2014				
2015					2015				
2016					2016				
2017					2017				
2018					2018				
2019					2019				
2020					2020				
Average data coverage									
<50%									
>50%									
