# Supplementary material for: Investigating the Variation of Benzene and 1,3-Butadiene in the UK during 2000–2020
Source: Int J Environ Res Public Health. 2022 Sep 21;19(19):11904. doi: 10.3390/ijerph191911904 (PMC9564389; doi:10.3390/ijerph191911904)

S1: Line plots of hourly benzene concentrations averaged per year with sufficient data availability for each of the four sites. Linear lines of best fit across entire dataset, along with appropriate equations have been imposed on the graphs.

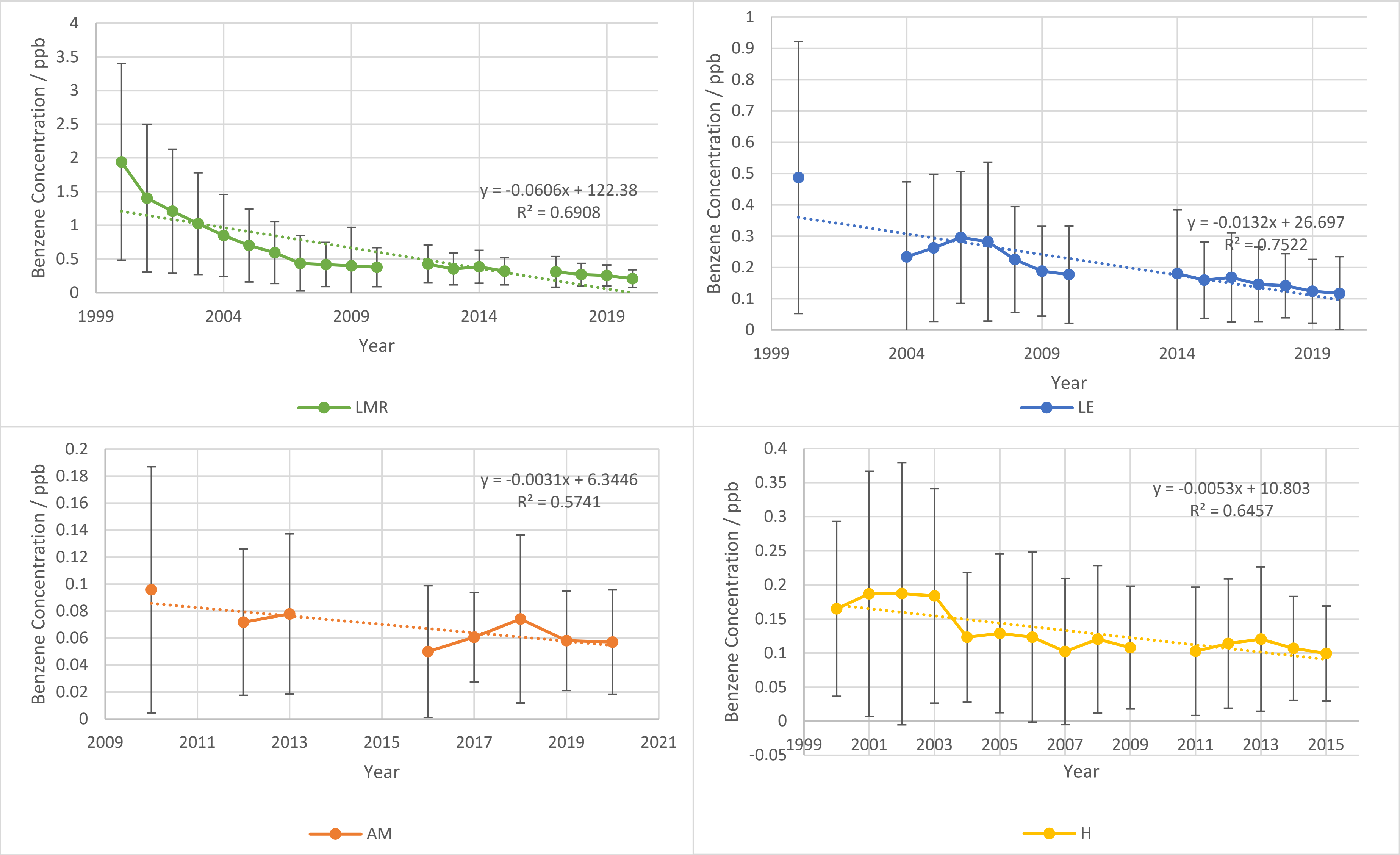

S2: Line plots of hourly 1,3-butadiene concentrations averaged per year with sufficient data availability for each of the four sites. Linear lines of best fit across entire dataset, along with appropriate equations have been imposed on the graphs.

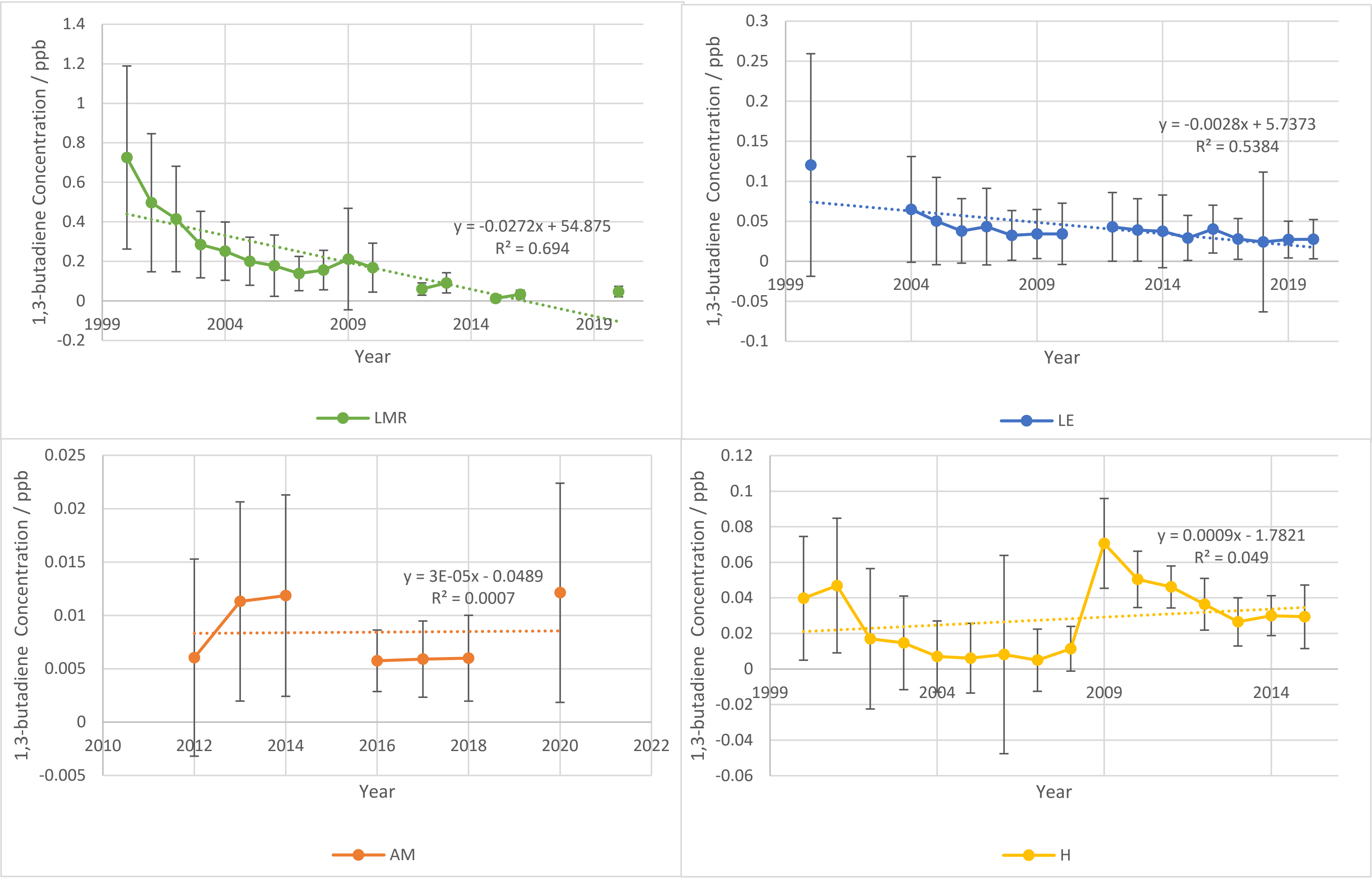

S3: Table presenting the relative statistical significance for each linear regression performed on dataset for each site and each pollutant

Site	Pollutant	p value	Statistical significance
**LMR**	Benzene	1.04E-05	$\rho < 0.05$
	1,3-butadiene	6.15E-05	$\rho < 0.05$
**LE**	Benzene	2.82E-05	$\rho < 0.05$
	1,3-butadiene	0.000800282	$\rho < 0.05$
**AM**	Benzene	0.029411926	$\rho < 0.05$
	1,3-butadiene	0.955889569	$\rho > 0.05$
**H**	Benzene	0.00030717	$\rho < 0.05$
	1,3-butadiene	0.41013216	$\rho > 0.05$

S4: Line plots of hourly benzene concentrations averaged per year with sufficient data availability for each of the four sites. Dataset has been split into two 10-year period (2000-2010, 2010-2020) where data is available. Linear lines of best fit across each decadal subsection, along with appropriate equations, have been imposed on the graphs.

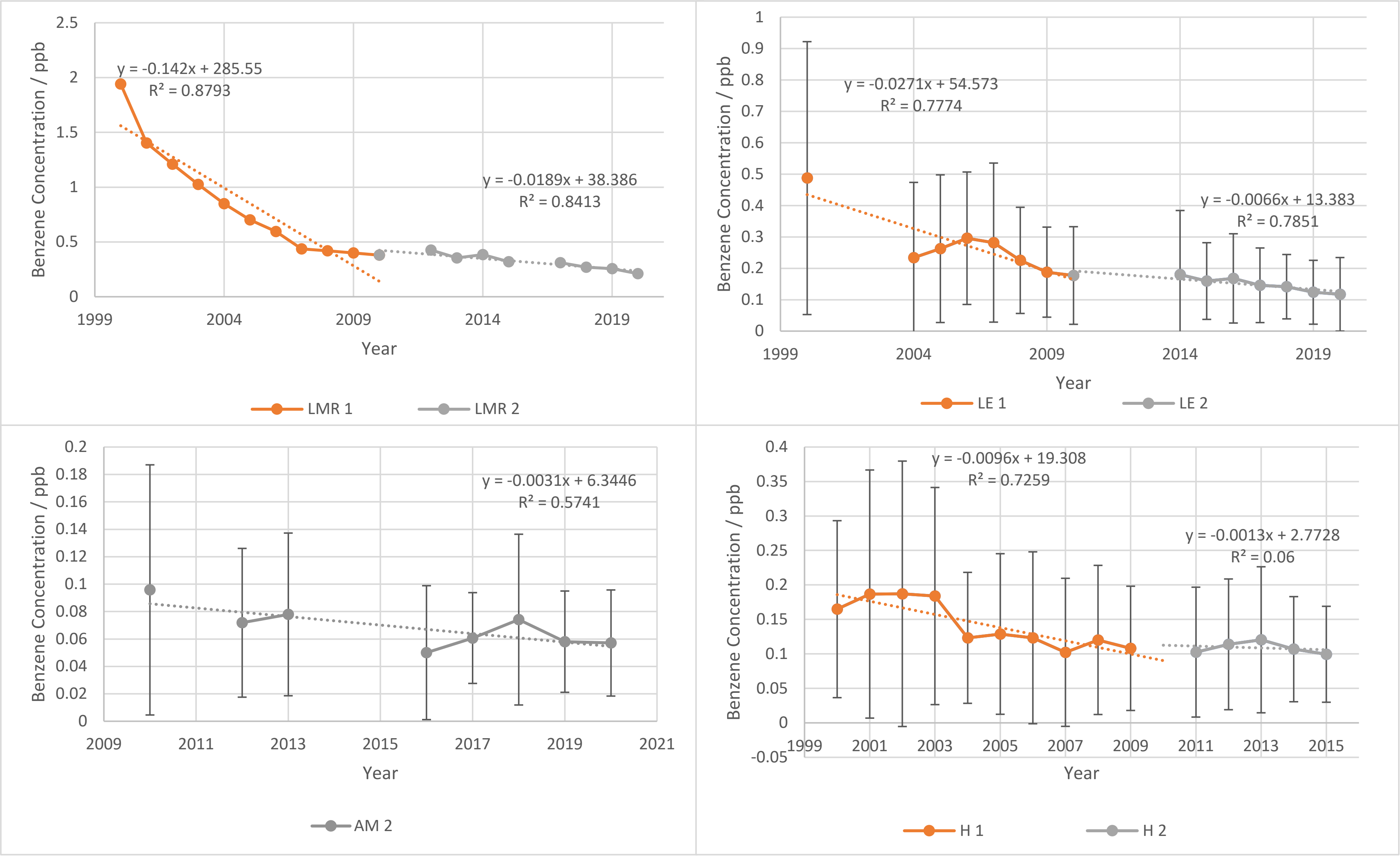

S5: Line plots of hourly 1,3-butadiene concentrations averaged per year with sufficient data availability for each of the four sites. Dataset has been split into two 10-year period (2000-2010, 2010-2020) where data is available. Linear lines of best fit across each decadal subsection, along with appropriate equations, have been imposed on the graphs.

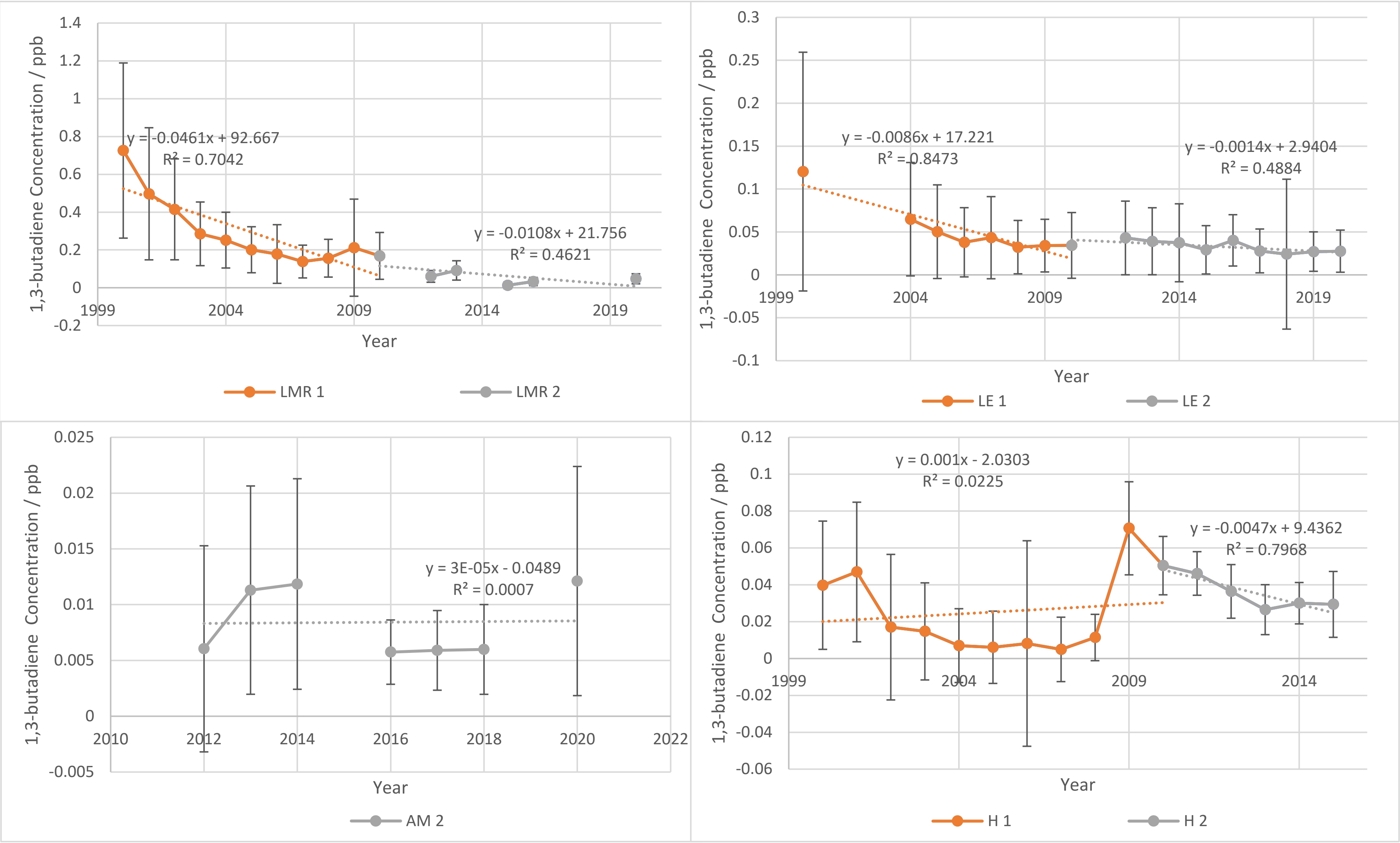

Supplement: Supplementary file 1 [file ijerph-19-11904-s001.zip › ijerph-1846324-supplementary.pdf]
